# Psychological Well-Being in Younger and Older Adults After Hurricane Ida

**DOI:** 10.1093/geroni/igaf122.1974

**Published:** 2025-12-31

**Authors:** Piper Bordes, Katie Cherry

**Affiliations:** Louisiana State University, Baton Rouge, Louisiana, United States; Louisiana State University, Baton Rouge, Louisiana, United States

## Abstract

Hurricane Ida slammed into the coast of south Louisiana on August 29, 2021 sixteen years to the day after Hurricane Katrina crippled the US Gulf Coast. Ida is considered the second most destructive hurricane known to the state of Louisiana following Katrina. The present study is part of a larger research program on risk and resilience factors in younger and older adults after the 2021 Hurricane Ida. In this study, we assessed symptoms of depression using the PHQ-9 and post-traumatic stress using the PCL-C in a sample of 128 community-dwelling adults. Half of the sample was recruited from three parishes (counties) where Ida damage was more severe and the other half was sampled from two parishes farther inland with less damage. All were tested individually, either in person or virtually via Zoom platform in a single session where they responded to individual difference measures, including state hope (agency and pathways subscales) and a structured storm questionnaire. Correlation analyses revealed that agency and pathways were negatively related to symptoms of depression and post-traumatic stress, while hurricane stressors and prior lifetime trauma were positively associated both mental health indicators. Regression analyses confirmed that only agency, pathways, and prior lifetime trauma significantly predicted depression symptoms, while agency and prior lifetime trauma significantly predicted symptoms of post-traumatic stress. Implications of these data for identifying positive factors that may promote long-term recovery and foster post-disaster psychological health will be discussed.

